# Comprehensive Analysis of Methyl-β-D-ribofuranoside: A Multifaceted Spectroscopic and Theoretical Approach

**DOI:** 10.1021/acs.jpca.4c00266

**Published:** 2024-03-12

**Authors:** Matei Pascariu, Leonardo Bernasconi, Matthew Krzystyniak, James Taylor, Svemir Rudić

**Affiliations:** † ISIS Neutron and Muon Source, Rutherford Appleton Laboratory, STFC, Harwell Campus, Chilton, Oxfordshire OX11 0QX, U.K.; ‡ Department of Chemistry, The University of Manchester, Oxford Road, Manchester M13 9PL, U.K.; § Center for Research Computing & Department of Chemistry, University of Pittsburgh, Pittsburgh, PA 15260, United States

## Abstract

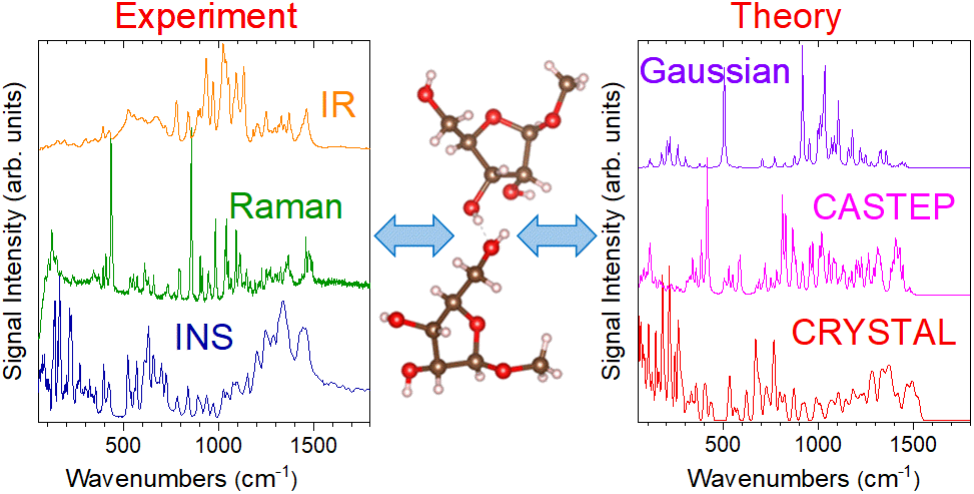

This study presents a comprehensive analysis of the vibrational spectra of methyl-β-D-ribofuranoside. Employing a combination of inelastic neutron scattering, Raman, and infrared spectroscopy allows for the observation of all modes regardless of the selection rules. The experimental techniques were complemented by density functional theory computational methods using both gas-phase (*Gaussian*) and solid-state (*CRYSTAL*, *CASTEP*) approaches to provide an unambiguous assignment of the defining vibrational features. Two distinct structures of the molecule were identified in the unit cell, differentiated mainly by the orientation of the furanose ring O–H bonds. The low-energy region of the spectrum (<400 cm^–1^) is dominated by lattice vibrations and functional group rotation, while the midenergy region is dominated by out-of-plane bending motions of the furanose ring (400–900 cm^–1^) and by C–H bending in the methyl and methylene groups (1400–1600 cm^–1^). The high-energy region (>2800 cm^–1^) encompasses the C–H and O–H stretching modes and offers convincing evidence of at least one H-bonding interaction between the two structures of methyl-β-D-ribofuranoside.

## Introduction

Furanosides, characterized by their five-membered ring structure known as a furanose ring, are molecules of great biological significance. Examples like ribose and deoxyribose are integral components of RNA and DNA, respectively, shaping their three-dimensional structures and biological functions.^1^ Beyond nucleic acids, furanosides are crucial in antibiotics, antiviral drugs, and other biologically active compounds, impacting their efficacy and interactions.^2^ Moreover, furanosides offer a compelling platform for studying molecular recognition and binding processes fundamental to biochemical interactions.^3,4^ The unique dynamics of furanose rings, including pseudo rotation and ring puckering, influences their behavior and interactions. These dynamic features are critical for understanding the structure–function relationship of furanosides and other biomolecules.^5,6^ The delicate balance between enthalpy and entropy governs these motions, making certain conformations thermodynamically favorable in biological contexts.

**Figure 1:**
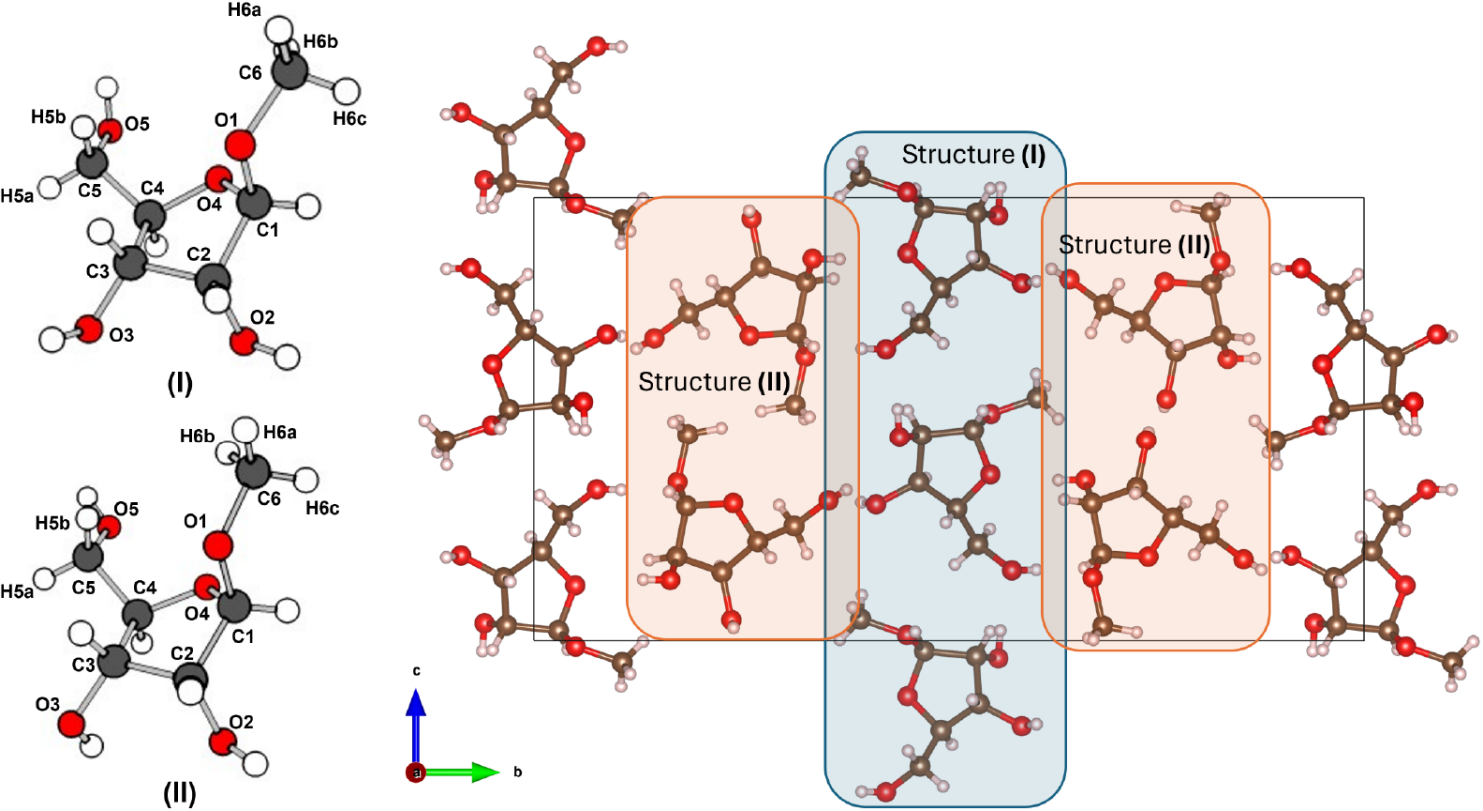
(left) The two distinct experimental structures found in the asymmetric unit cell of methyl-β-D-ribofuranoside, labeled with the convention used for the rest of the work. The structures have been manually selected from the crystallographic information file (CIF) deposited as part of ref (16). (right) View along the *x*-axis of the asymmetric unit cell of methyl-β-D-ribofuranoside. The two distinct structures are highlighted, with alternating rows of structure **(I)** molecules (blue, middle) and structure **(II)** molecules (orange, left and right).

Methyl-β-D-ribofuranoside (Figure 1), a representative of furanosides, plays a vital role in various natural compounds and biochemical processes. This molecule has garnered scientific interest due to its structural and dynamical properties.^7−14^ While X-ray and neutron diffraction has provided valuable structural insights,^15−17^ nuclear magnetic resonance spectroscopy has probed its conformations in solution.^18−20^ Vibrational spectroscopy is an essential tool for probing the structure and dynamics of complex molecules,^21−23^ providing insights into bonding interactions, functional groups, conformation, and molecular environment. For studying carbohydrates in particular, Raman spectroscopy and Raman optical activity measurements are state of the art techniques which exploit the chirality of the molecules to establish structural characteristics.^24^ To the best of the authors’ knowledge, vibrational data in the literature of methyl-β-D-ribofuranoside are limited,^25,26^ which is perhaps why there remains a gap in understanding the vibrational dynamics of this molecule.^27−32^ Hence, the present study aims to report and analyze the vibrational profiles of the molecule utilizing three spectroscopic techniques: inelastic neutron scattering (INS), Raman, and infrared (IR) spectroscopy. The spectroscopic techniques are augmented with computational methods, primarily density functional theory (DFT), offering insights into the molecule’s energy landscape, pseudo rotation, and puckering effects. Starting from the structural studies published by C. A. Podlasek^16^ and A. G. Evdokimov,^17^ this study diverges from the conformational analysis of methyl-β-D-ribofuranoside and aims to establish a consistent methodology of extracting information from various experimental sources and simulated data. At the same time, this study reports spectroscopic data that were not available before for this system. The unique combination of vibrational spectroscopic techniques and computational approaches allows one to explore the intricate behavior of methyl-β-D-ribofuranoside, bridging the gap in the understanding of this important class of compounds.

## Methods

### Experimental Spectroscopy

The methyl-β-D-ribofuranoside sample was used as supplied by Sigma-Aldrich as a crystalline solid, without undergoing any intervention. For each experimental technique, a small quantity of the sample from the same batch was loaded following the procedures specific for each instrument, which are described, where necessary, in the associated references.

High-resolution inelastic neutron scattering (INS) experiments were carried out using a TOSCA spectrometer,^33,34^ situated at the ISIS Pulsed Neutron & Muon Source located in Oxfordshire, UK. The INS technique proves highly effective in elucidating a comprehensive spectrum encompassing all vibrational modes within the entire range of interest, extending up to approximately 500 meV (equivalent to 4000 cm^–1^) and with spectral resolution of approximately 1.25% *ET* i.e., a fixed percentage of the energy transfer. The experimental data obtained on TOSCA was processed and reduced using the Mantid software.^35,36^

A distinct advantage of employing INS over traditional optical methods, such as infrared (IR) or Raman spectroscopy, stems from the absence of optical selection rules, as all vibrational transitions are inherently accessible. This aspect holds particular significance in unraveling intricate molecular behaviors. INS exhibits distinct sensitivity toward hydrogen modes, setting them apart from other types of atomic displacements found within complex molecules. This feature is attributed to the comparatively large incoherent neutron cross-section exhibited by hydrogen nuclei, rendering their vibrational patterns particularly discernible through INS measurements.

Two experimental Raman setups were used for the study of methyl-β-D-ribofuranoside: a custom setup using a Renishaw inVia Raman spectrometer and a commercial Bruker Senterra Raman microscope. The customized Raman setup was designed to achieve measurements at cold temperatures, matching the conditions of the INS experiments. It consists of a custom-made center stick, suitable for insertion into a 100 mm-bore cryostat, which allows for measurements at temperatures between 4 and 350 K, to which a laser probe head fiber optically coupled to a Renishaw inVia Raman spectrometer can be attached. The setup offers a choice of two lasers: a 532 nm, 200 mW, Class 3B, continuous wave, diode-pumped solid-state laser or a 785 nm, 300 mW, Class 3B, continuous wave, Toptica diode-pumped solid-state laser. Overall, the setup enables the measurement of the Raman spectra of solid samples with a resolution of 1 to 4 cm^–1^ over a wide range (0–4000 cm^–1^). More details about this setup can be found in the Supporting Information (SI) and in a work by M. A. Adams et al.^37^ For comparison purposes, Raman spectra were also recorded using a commercial benchtop Bruker Senterra confocal Raman microscope setup, equipped with lasers of three different wavelengths: 532, 633, and 785 nm and capable of measurements of similar resolution (3 to 5 cm^–1^) and range (40–4000 cm^–1^). The Raman spectra of methyl-β-D-ribofuranoside were recorded using the 532 and 785 nm wavelength lasers at a power of 5.0 mW.

The infrared (IR) spectroscopy measurements were performed under normal atmospheric conditions at room temperature, using a Bruker VERTEX 70v Fourier Transform IR (FTIR) interferometer. For the IR spectroscopy results, the background was first measured for 128 scans, and then, the spectra were acquired for 64 scans at a resolution of 4 cm^–1^. When loading, the sample was placed on top of a diamond crystal and held in position by pressure applied via a clamp mechanism.

### Computational Details

The *Gaussian 16*(38) software was used for gas-phase simulations, while for solid-state calculations, the C*RYSTAL 17*^39^ and *CASTEP 23*(40) codes were employed. The fundamental difference between *CRYSTAL* and *CASTEP* stems from the type of basis sets used in the expansion of the electron density and the DFT one-particle orbitals, with the former employing Gaussian-type basis sets and the latter plane-wave basis sets. Each approach has its own benefits and limitations; thus, combining both offers a more accurate and versatile analysis. The structural basis for the calculations was the X-ray diffraction structure of methyl-β-D-ribofuranoside deposited by C. A. Podlasek et al.^16^ as a CIF in the Cambridge Structural Database (CSD). The simulation procedure was the same for all employed codes: the geometry was optimized first, then, the frequencies of the vibrational modes were calculated, and their intensities were simulated to yield INS, Raman, and IR spectra. The INS spectra were simulated with the AbINS plugin,^41^ available in Mantid, which used the phonon data calculated with each code to which it applied the energy-dependent resolution function specific to each instrument (TOSCA in this case). The Raman and IR intensities were also simulated using the specific keywords for each code (the input files are available in the SI), and the respective spectra generated with the Chemcraft^42^ software where a Lorentzian broadening of 8 cm^–1^ has been applied for an easier comparison with the experimental data. Where possible, hybrid functionals were opted for because of their improved accuracy compared to Local Density Approximation (LDA) and Generalized Gradient Approximation (GGA) functionals.^43−46^

In the single-molecule DFT simulation in *Gaussian 16*, the starting geometry was given by the coordinates of a single molecule of each of the two distinct structures selected from the referenced CIF. The convergence criteria for the geometry optimization were formulated in terms of the root mean squares (RMSs) on force gradient and the RMS atomic displacement. The optimization stopping criteria were set to 0.0003 au in the case of the RMS on force gradient and 0.0012 au for the RMS atomic displacement. The hybrid density functional B3LYP^47^ was employed. Additionally, in order to account for the nonhomogeneity of the electron density distribution in the system, the expansion was employed in terms of the density gradient (referred to as generalized gradient approximation, GGA). The GGA-type functional PBEPBE, which is the *Gaussian* implementation of the local functional PBE,^48^ was used for the DFT simulation, together with the 6-311++G(d, p) basis set.

For the solid-state DFT simulation in *CRYSTAL 17*, the input geometry was given by the atomic coordinates of the asymmetric unit cell from the referenced CIF. These were then optimized, keeping the cell parameters constant. The convergence criteria for the geometry optimization were formulated in terms of the RMS on force gradient and the RMS atomic displacement. The optimization stopping criteria were set to values ten times smaller than those for the single-molecule simulation in *Gaussian*, with an RMS on force gradient of 0.00003 au and an RMS on atomic displacement of 0.00012 au. The B3LYP and PBESOL0 hybrid density functionals were used. PBESOL0 is the solid-state adaptation of the hybrid exchange-correlation functional PBE0,^49^ which, in turn, is the hybrid adaptation of the local functional PBE. The basis set used was a solid-state adaptation of conventional molecular Gaussian basis sets, and the exponents and contraction coefficients used to define it are given in the SI. The Brillouin zone was sampled using a 4 × 4 × 4 Monkhorst–Pack grid.

For the solid-state DFT simulation in CASTEP 23, the procedure was the same as that for the *CRYSTAL* simulation, with the same input geometry. The cell parameters were again kept constant, and only the atomic coordinates were allowed to change in order to yield an optimized structure. Exchange and correlation were approximated using the solid-design version of the PBE functional, PBESOL.^50^ The Tkatchenko–Scheffler dispersion correction scheme within the generalized gradient approximation and on-the-fly generated (OTFG) norm-conserving pseudopotentials were used. The plane-wave cutoff energy was 1050 eV, and the Brillouin zone sampling of electronic states used a 4 × 1 × 2 Monkhorst–Pack grid.

## Results and Discussion

### Structural Analysis

The crystal of methyl-β-D-ribofuranoside (C_6_H_12_O_5_, FW 164.16) is orthorhombic (space group *P2*_*1*_*2*_*1*_*2*_*1*_) with eight molecules in the unit cell (*Z* = 8), based on the experimentally determined X-ray structure^16^. The cell parameters are *a* = 4.8595 Å, *b* = 24.162 Å, *c* = 12.876 Å, and α, β, γ *=* 90°, with a cell volume of *V* = 1511.84 Å^3^. The crystallographic unit cell contains two distinct molecular structures,^17^ labeled **(I)** and **(II)** in Figure 1. The molecules of each structure are arranged linearly along the *z*-axis with alternating opposite orientations of each molecule along the line. Following the *y*-axis, the rows are formed of alternating structure **(I)** and structure **(II)** molecules. The major difference between the two structures is given by the C3–O3 bond, and it can be quantified by looking into the values of the experimental torsion angle τ (H, O3, C3, H). While for structure **(I)**, this torsion angle has a value of −17.50° (±0.2°), in structure **(II)** the O3–H group is pointing almost in the opposite direction, with a torsion angle of 179.66° (±0.2°).

As part of the structural analysis, a detailed comparison between experimental and optimized structures has been conducted, focusing on key parameters such as bond lengths and torsion angles (see Tables 1 and 2). All the other structural parameters can be obtained from the CIFs provided as part of the SI. To enable a comprehensive assessment of structural changes, torsion angles have been opted for as they provide concise information about bond rotations and twists in the optimized geometry molecules and can easily uncover distortions from the experimentally determined structures. The notation used for atom labeling was consistent with Figure 1.

**Table 1:** Selected Experimental and Computed Structural Parameters (Bond Lengths and Torsional Angles are Given in Units of Å and deg, Respectively) for the Methyl-β-D-ribofuranoside Structure (I)^a^

		*CRYSTAL*		*Gaussian*	
	expt. X-ray^b^	B3LYP	PBESOL0	B3LYP	PBEPBE
*r* (C1–H)	0.934	1.097	1.102	1.095	1.105
*r* (C2–H)	0.974	1.095	1.102	1.094	1.105
*r* (C3–H)	0.981	1.096	1.102	1.094	1.104
*r* (C4–H)	0.926	1.098	1.102	1.095	1.105
*r* (C5–H5a)	0.931	1.098	1.103	1.100	1.110
*r* (C5–H5b)	0.987	1.097	1.103	1.098	1.107
*r* (C6–H6a)	0.955	1.093	1.097	1.090	1.098
*r* (C6–H6b)	0.936	1.094	1.099	1.094	1.103
*r* (C6–H6c)	0.952	1.098	1.103	1.098	1.107
*r* (O2–H)	0.848	0.983	0.985	0.967	0.978
*r* (O3–H)	0.842	0.984	0.984	0.962	0.971
*r* (O5–H)	0.850	0.985	0.987	0.961	0.970
τ (H,C1,O1,C6)	41.24	39.82	40.98	53.38	53.70
τ (H,C4,C5,H5a)	65.05	65.35	64.90	71.84	71.17
τ (H,C4,C5,H5b)	–179.50	–176.62	–177.58	–169.98	–171.11
**τ (H,O2,C2,H)**	**–44.62**	**–48.98**	**–51.26**	**96.84**	**100.63**
τ (H,O3,C3,H)	–17.50	–23.15	–17.80	–31.04	–33.37
**τ (H,O5,C5,H5a)**	**112.68**	**110.95**	**115.01**	**67.33**	**67.11**
**τ (H,O5,C5,H5b)**	**–5.45**	**–8.92**	**–4.72**	**–53.89**	**–54.39**

a Significant differences are given in bold

b Average estimated standard deviation (AESD) for experimentally determined parameters: 0.003 Å on bond distances, 0.2° on torsion angles.

**Table 2:** Selected Experimental and Computed Structural Parameters (Bond Lengths and Torsional Angles are Given in Units of Å and deg, Respectively) for Methyl-β-D-ribofuranoside Structure **(II)**

		*CRYSTAL*		*Gaussian*	
	expt. X-ray^a^	B3LYP	PBESOL0	B3LYP	PBEPBE
*r* (C1–H)	0.937	1.095	1.101	1.098	1.108
*r* (C2–H)	0.992	1.096	1.102	1.091	1.101
*r* (C3–H)	0.968	1.095	1.101	1.092	1.102
*r* (C4–H)	0.947	1.097	1.103	1.095	1.105
*r* (C5–H5a)	0.964	1.099	1.105	1.099	1.109
*r* (C5–H5b)	1.032	1.099	1.104	1.098	1.108
*r* (C6–H6a)	0.935	1.092	1.096	1.089	1.098
*r* (C6–H6b)	0.999	1.094	1.097	1.093	1.102
*r* (C6–H6c)	0.926	1.100	1.104	1.098	1.107
*r* (O2–H)	0.802	0.978	0.979	0.961	0.970
*r* (O3–H)	0.743	0.983	0.984	0.967	0.977
*r* (O5–H)	0.787	0.978	0.979	0.961	0.970
τ (H,C1,O1,C6)	58.15	56.46	55.78	55.45	55.56
τ (H,C4,C5,H5a)	65.98	64.42	67.43	72.20	71.80
τ (H,C4,C5,H5b)	–172.51	–178.51	–176.90	–169.79	–170.65
τ (H,O2,C2,H)	–29.99	–46.77	–46.47	–43.09	–39.91
τ (H,O3,C3,H)	179.66	175.42	173.38	–159.69	–159.07
τ (H,O5,C5,H5a)	69.75	57.29	60.99	63.04	62.52
τ (H,O5,C5,H5b)	–55.20	–62.36	–58.64	–58.19	–58.98

a Average estimated standard deviation (AESD) for experimentally determined parameters: 0.003 Å on bond distances, 0.2° on torsion angles.

Our first observation is that a consistent trend is observed: optimized bonds tend to be longer than their experimentally determined counterparts, which was noted in previous studies of furanosides.^19^ This disparity between experiment and theory is particularly pronounced for the O–H and C–H bonds compared to the C–C and C–O bonds of the furanose ring, which is why only the former were considered in the paper. In terms of the parameters of the calculations, all of the bonds simulated with PBE-based functionals (the hybrid solid-state adaptation PBESOL0 and the GGA-type functional PBEPBE) are longer than the ones obtained from simulations employing B3LYP. The difference is slightly larger in the single-molecule simulation, with the GGA-type functional PBEPBE yielding the most significant deviation from the experimental results between all of the functionals used in the study.

In the analysis of structure **(I)** of methyl-β-D-ribofuranoside (see Table 1), the single molecule optimization with the B3LYP hybrid functional resulted in O–H and C–H bond length values that were closer, in general, to the experimental values than in the case of solid-state simulations with the same functional. However, the difference is arguably small between the bond lengths optimized in *CRYSTAL* and *Gaussian*, especially in the case of C–H bonds. Notably, on the other hand, torsion angles revealed significant deviations. The O2–H bond of structure **(I)** shifted inward in the *Gaussian* optimization at a torsion angle of 96.84° (B3LYP)/100.63° (PBEPBE) with the corresponding C2–H bond plane compared to the −44.62° in the experimental structure. The stabilizing factor, in this case, is the possibility of forming an intramolecular H–bond with O3, in contrast with the crystal structure where the O2–H bond points outward, suggesting the presence of stabilizing interactions through this specific bond with neighboring molecules in the solid state. Differences of almost 50° from the experimental data are also observed in the torsion angles involving the O5–H bond of the *Gaussian*-optimized structure. Similar features are observed in the parameters of methyl-β-D-ribofuranoside structure **(II)** (see Table 2), with gas-phase simulations yielding more accurate C–H and O–H bond length values when the hybrid B3LYP functional is used but overestimating the C–H bond lengths, in particular, with the PBEPBE functional. This is in comparison to the solid-state simulations, given that all of the simulated parameters are higher in value than the experimental data.

At this point, fundamental distinctions between the single molecule and crystal structure optimizations need to be addressed. Gas-phase DFT calculations treat molecules in isolation without a proper representation of the molecular environment; therefore, the resulting minimum energy structure only highlights possible intramolecular interactions. In contrast, solid-state simulations consider the crystal environment and the influence of intermolecular interactions explicitly, leading to geometric adjustments that reflect the effects of the crystal lattice on the molecule. The choice of DFT functional and basis set plays a pivotal role in determining bond lengths and angles, as certain functionals may better account for specific interactions, such as dispersion forces, commonly found in solid-state systems, and hydrogen bonds, which are crucial in describing the bulk properties of matter in a variety of systems. It is important to note that such comparisons offer qualitative insights into structural stabilization (or destabilization) in molecules. These optimizations were a prerequisite for further vibrational mode calculations, but a more substantial comparison between the employed density functionals and basis sets is based on the agreement between experimental and simulated vibrational spectra. The structural analysis, as a first impression, underscores these distinctions: both solid-state and single-molecule optimizations align with X-ray diffraction data obtained from crystalline samples, while deviations from experimental geometry are evident only in bonds where distinct intermolecular (for solid-state) or intramolecular (for gas-phase) interactions could be expected. This aspect will be further investigated with a more detailed analysis of the vibrational data.

### INS Data Analysis

**Figure 2:**
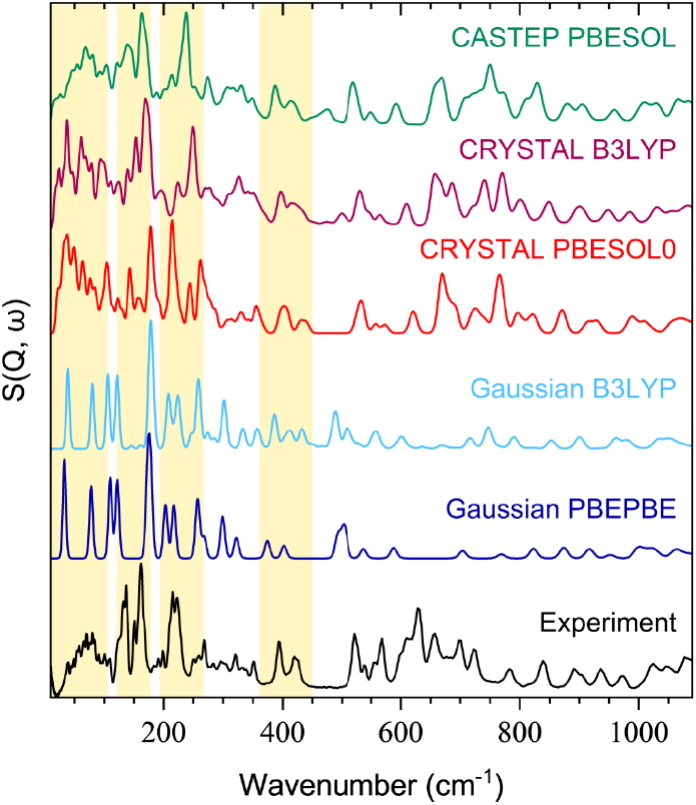
Comparison in the low-energy region of the experimental INS spectrum of methyl-β-D-ribofuranoside with theoretical spectra simulated with *Gaussian* (gas-phase approach, using hybrid B3LYP and GGA-type PBEPBE functionals), *CRYSTAL* (solid-state approach, using hybrid B3LYP and PBESOL0 functionals), and *CASTEP* (solid-state approach, using the GGA-type PBESOL functional).

The TOSCA neutron spectrometer is optimized for high-resolution analysis in the lower-energy region, facilitating the separation of fingerprint signals and enabling the study of modes below 400 cm^–1^, which are typically challenging to access using Raman and IR spectroscopy. These modes hold critical information about lattice vibrations and intricate large-scale motions within the unit cell, crucial for crystal structure investigations. The comparative analysis of INS spectra (Figure 2), both experimental and generated through DFT simulations, reveals that solid-state simulations offer superior accuracy, especially in describing vibrations below 400 cm^–1^. Simulations performed on a single molecule using *Gaussian* exhibit a simpler pattern, with several peaks absent compared to those in experimental and solid-state simulated spectra. *CRYSTAL* simulations, on the other hand, capture intensity patterns with satisfactory overall agreement. An important aspect to note at this moment is that all of the single-molecule simulated spectra presented in this study are for structure **(I)** of methyl-β-D-ribofuranoside. This is done in order to avoid unnecessarily complicating the figures and to ease analysis of the features. While there are differences expected between the simulated spectra of the two structures, the *Gaussian* simulation is used in this instance to facilitate the identification of the vibrational modes and any inaccuracies of the single-molecule calculations are addressed by using the solid-state approaches in *CRSYTAL* and *CASTEP*. When considering the density functionals employed for each method, the differences are not as straightforward to interpret. Although arguments in favor of one functional over the other will be highlighted throughout the discussion of the relevant spectral features, the aim is not to decide on the “best” exchange-correlation approximation, as such categorization may be counterproductive for further studies, but rather to assess what can be achieved by comparing each approach.

As emphasized throughout this study, the low-energy spectrum in solids is dominated by lattice vibrations, encompassing collective atomic motions within the crystal lattice. Additionally, rotational and translational motions, peak overlap due to similar energies, and computational challenges related to long-range interactions may contribute to the complexity of the spectral response in this region.^51^ Below 20 cm^–1^ in the experimental spectrum, the elastic peak prevails, while the solid-state simulation reveals translational motions along the *x*, *y*, and *z* directions, all at 0 cm^–1^. Between 20 and 115 cm^–1^, overlapping peaks and relatively low intensities present analytical challenges. However, the solid-state calculations offer valuable insights into the types of motions occurring. In the *CRYSTAL* data, there are 45 modes in this range, all of which can be described as collective, indicating contributions from the entire crystalline lattice of methyl-β-D-ribofuranoside. The first three modes (18 cm^–1^, 24 cm^–1^, and 25 cm^–1^) involve shear-like motions in different directions, followed by collective rotations of molecular units (31 to 49 cm^–1^), causing lattice deformations. Beyond 56 cm^–1^, vibrations introduce furanose ring deformations, with modes up to 114 cm^–1^ characterized by ring distortions that bring substituents closer together in a folding motion. In contrast, gas-phase simulations identify only three modes (40 cm^–1^, 81 cm^–1^, and 108 cm^–1^) exhibiting similar motions with slight furanose ring deformations.

After 115 cm^–1^ in the experimental spectrum, there are at least six visible modes (including the shoulders of the stronger peaks) that end at 175 cm^–1^. The simulation done with the *CRYSTAL* code lists 24 modes in this region, all being complex distortions that lead to significant displacements of methyl and hydroxymethyl groups in a rotation-like motion. The single molecule simulation identifies a similar motion of the atoms but only as two vibrational modes, at 123 and 178 cm^–1^. The next interesting features are the two strong peaks at 214 and 227 cm^–1^ in the experimental spectrum. On this occasion, the simulation done with the *Gaussian* code matches the data, with two peaks at 208 and 224 cm^–1^, suggesting the rotation of the C–O bonds as the cause of these modes. The peaks between 375 and 450 cm^–1^ also match, this time with the solid-state simulation as well. Here, in-plane and out-of-plane deformations of the furanose ring start to appear. From this point upward on the energy scale, the Raman data are easier to analyze and more conclusive.

The INS spectrum was also simulated with the *CASTEP* code. The reason here was to compare the accuracy of the plane-wave basis sets to the Gaussian-type basis sets used for the simulated data presented up to this point. As for the previously presented results, the spectra agree overall with certain differences in regions with several overlapping peaks, such as the signals between 600 and 800 cm^–1^, where there is a significant shift between the theoretical and experimental peaks. The most interesting region, however, is the low-energy fingerprint zone. Of all the simulated spectra, the one from the *CASTEP* calculation shows the best match, with a similar intensity pattern up to 200 cm^–1^.

### Raman Data Analysis

**Figure 3:**
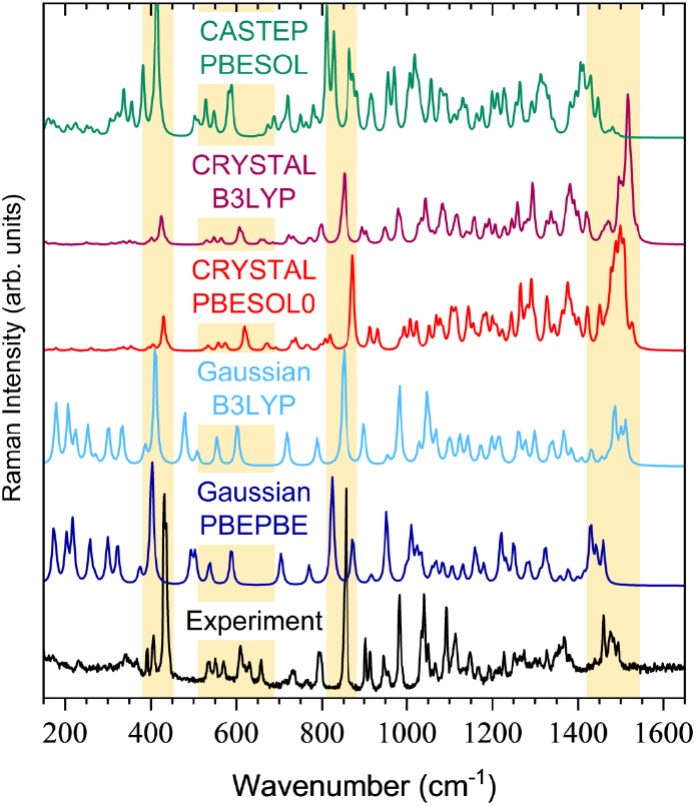
Comparison in the intermediate-energy region of the experimental Raman spectrum of methyl-β-D-ribofuranoside with theoretical spectra simulated with *Gaussian* (gas-phase approach, using hybrid B3LYP and GGA-type PBEPBE functionals), *CRYSTAL* (solid-state approach, using hybrid B3LYP and PBESOL0 functionals), and *CASTEP* (solid-state approach, using the GGA-type PBESOL functional).

For the Raman spectra, the simulations done using the B3LYP hybrid exchange-correlation functional were overall more accurate than those with the PBEPBE/PBESOL0 functionals in terms of peak positions and intensity patterns. A prominent comparison is presented in Figure 3, revealing a generally good overall agreement, particularly favoring the solid-state simulation, as expected. While some discrepancies are evident in regions with complex intensity patterns and overlapping peaks, the most pronounced and well-defined modes demonstrate close alignment between the theoretical and experimental data. For instance, the two strong peaks at 442 and 855 cm^–1^ are found both in the solid-state (437 and 854 cm^–1^) and gas-phase (411 and 851 cm^–1^) simulations and correspond to out-of-plane bending motions of the furanose ring, exhibiting high symmetry and intense vibrational activity.

On the other hand, a significant divergence emerges in the fingerprint region (150 to 390 cm^–1^). While solid-state simulations in *CRYSTAL* show either very weak or inactive Raman peaks in this region, gas-phase simulations identify nine Raman-active frequencies for the isolated molecule. These modes involve intricate combinations of in-plane and out-of-plane deformations of the furanose ring and rotations around C–C and C–O bonds, reflecting their complex nature. The low intensity of these vibrations in the experimental data could be explained by the inherent asymmetry of the composite vibrations present at this energy level, which is, nonetheless, captured in the solid-state simulation. Further analysis delves into the 500–700 cm^–1^ region, where solid-state simulations match experimental data, portraying in-plane bending of the ring caused by O–C and O–H bond angle contractions. Conversely, gas-phase simulations exhibit fewer modes that are significantly shifted. Specifically, the peak at 605 cm^–1^, common to both simulations, is followed by two weaker peaks in experimental data (637 and 663 cm^–1^), absent from the gas-phase simulation but present, albeit with slight shifts, in the solid-state simulation. These differences arise due to a degree of vibrational mode mixing and are attributed to motions such as twisting of the C–O bonds outside the furanose ring, affecting O–H bonds and hydrogen bonding, phenomena facilitated by intermolecular interactions present in the solid state. The grouping of peaks in the 1450–1550 cm^–1^ region is slightly shifted in both gas-phase and solid-state simulations. These peaks are linked to asymmetric C–H bending (scissoring) in the methyl and methylene groups, with the solid-state calculations also involving significant O–H bending motions. This interplay influences the intensity pattern, differentiating it from experimental data and the gas-phase simulation.

Additionally, the Raman spectrum was simulated using the *CASTEP* code, yielding results comparable to those obtained using *CRYSTAL*, although a notable shift occurs in the strong peak at 860 cm^–1^. Again, similar to the *CRYSTAL* INS solid-state simulations, the data obtained with *CASTEP* provide a more accurate depiction of the low-energy region, mirroring the intensity pattern closely.

### IR Data Analysis

**Figure 4:**
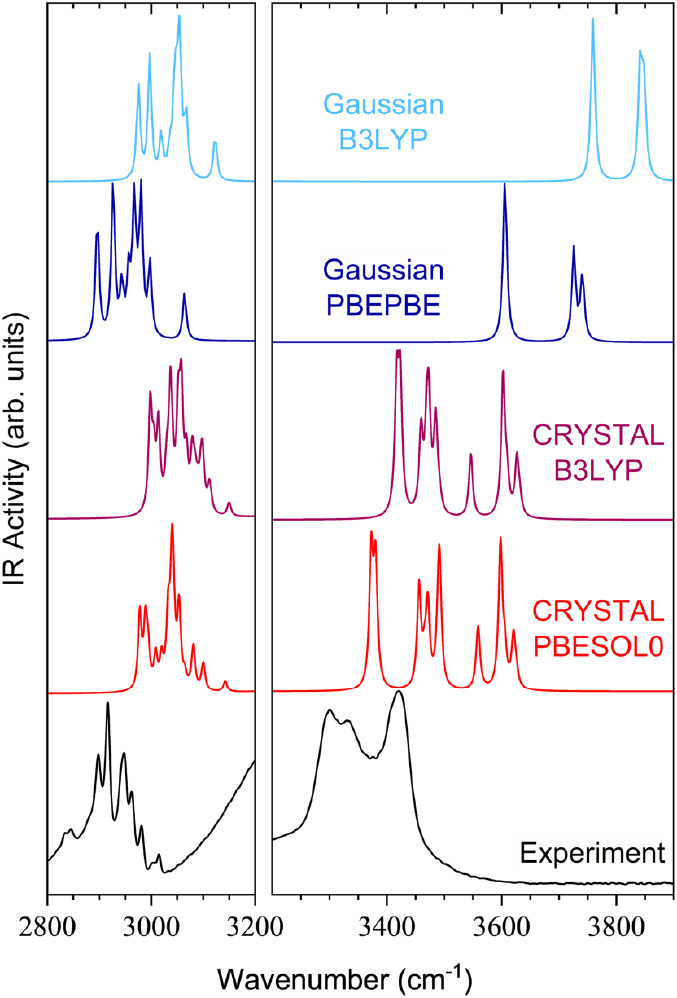
Comparison in the high-energy region of the experimental IR spectrum of methyl-β-D-ribofuranoside with theoretical spectra simulated with *CRYSTAL* (solid-state approach, using hybrid B3LYP and PBESOL0 functionals) and *Gaussian* (gas-phase approach, using hybrid B3LYP and GGA-type PBEPBE functionals). The intensities in the left panel (focusing on C–H vibrational modes) have been scaled to match more intense peaks in the right panel (focusing on the O–H stretching peaks) for a clearer comparison.

In our exploration of infrared spectroscopy of methyl-β-D-ribofuranoside, we primarily focused on the analysis of high-energy C–H and O–H stretching modes, an area where Raman and INS spectroscopy tend to provide limited resolution. On this instance, the simulations utilizing the PBE-based functionals exhibited better alignment with experimental data, despite an overall significant shift between computational and experimental results, as depicted in Figure 4. The C–H and O–H stretching modes, being inherently sensitive to experimental conditions and neighboring molecular interactions, as well as reliant on the choice of theoretical framework and dispersion correction coefficients, exhibited expected challenges in precise overlap between simulation and experimental data. Nevertheless, due to the fewer peaks and improved separation in this spectral region, a more intricate description of these vibrational modes could be provided. In the range of 2800 to 3200 cm^–1^, the single molecule simulation in *Gaussian* outperformed in describing C–H modes, while the solid-state simulation in *CRYSTAL* better represented the complex O–H modes, which were challenging to distinguish in the experimental spectrum.

The use of the GGA-type PBEPBE functional in the gas-phase simulation resulted in the closest match in peak positions between simulation and experiment in the C–H region; thus, a detailed analysis of the modes in this calculation will be provided. Nine C–H stretching modes were identified, each corresponding to the nine C–H bonds within a single molecule of methyl-β-D-ribofuranoside. The two primary peaks at 2895 and 2926 cm^–1^ were attributed to symmetric stretching of the C–H bonds in the C5 methylene and C6 methyl groups, respectively. Subsequently, five peaks (2943 cm^–1^, 2957 cm^–1^, overlapping 2967 and 2972 cm^–1^, 2980 cm^–1^) were assigned to asymmetric composite vibrations of all C–H bonds, followed by two peaks at 2997 and 3064 cm^–1^ representing the asymmetric stretching of the methyl group. For completion, the O–H stretching region featured three peaks, at 3606 cm^–1^ (O2–H), 3726 cm^–1^ (O3–H), and 3741 cm^–1^ (O5–H), respectively.

In the solid-state simulation, the complexity increased, with 72 C–H and 24 O–H stretching modes for the entire unit cell, eight times as much as for the gas-phase simulation. This observation, of course, aligns with the fact that there are eight molecules in the unit cell of the crystal. The C–H stretching modes show a pattern similar to the ones in the single molecule simulation. According to the PBESOL0 simulation, the first modes (2978–3009 cm^–1^) correspond to symmetric stretching of the C–H bonds of the C5 methylene and C6 methyl groups. Following are asymmetric composite vibrations of all the C–H bonds (3020–3081 cm^–1^). The last C–H modes (3099–3143 cm^–1^) are assigned to the asymmetric stretching of the methyl group. In the O–H region, there are notable differences from the single molecule simulation besides the number of modes. The lowest energy four O–H stretching vibrations (3373–3380 cm^–1^) are of the asymmetric stretching of the O5–H bonds of structure **(I)** molecules, with very weak contributions from other O–H bonds, as opposed to the single molecule simulation where the lowest energy O–H stretch was of the O2–H bond. The next four modes (3456–3466 cm^–1^) are the symmetric stretching motions of the same O5–H bonds of structure **(I)** molecules. Following are two overlapping asymmetric O–H stretches of the O3–H bonds of structure **(II)** molecules (3471.300 and 3471.303 cm^–1^). The next four modes (3489–3491 cm^–1^) are particularly interesting as they show composite asymmetric stretching of the O5–H bonds of structure **(II)** molecules and of the O3–H bonds of structure **(I)** molecules. Here is the clearest evidence for specific H-bonding in the crystal of methyl-β-D-ribofuranoside: the optimized structure suggests a geometry of the molecules such that the O3**(I)**–H–O5**(II)** H-bond is possible, but there is also direct vibrational evidence through these individual modes.

These observations highlight how the ribofuranoside molecule responds to its surrounding environment within the crystal lattice. Understanding these structural modifications and the differential sensitivity of various functional groups to interactions is crucial for discussions not only in the context of the crystal state but also in biological environments, particularly in aqueous solutions where hydrogen bond effects play a significant role. Finally, the highest energy ten modes (3559–3626 cm^–1^) are the results of composite stretching of all the O–H bonds, and no intricacies were observed in this region.

## Conclusions

This study presents a thorough analysis of the vibrational spectra of methyl-β-D-ribofuranoside, employing a combination of INS, Raman, and IR spectroscopy techniquies complemented by DFT calculations. Hybrid exchange-correlation functionals were employed in solid-state simulations using the *CRYSTAL 17* code (B3LYP and PBESOL0) and the gas-phase approach with the *Gaussian 16* code (B3LYP), alongside GGA-type functionals for the solid-state simulation in *CASTEP 23* (PBESOL) and the single molecule simulation in *Gaussian16* (PBEPBE). While one of the aims was to compare the density functionals, no clear preference for accuracy emerged, as both yielded comparable results for geometry optimization and vibrational spectral simulations. In the case of INS spectroscopy, B3LYP proved to be more suitable in the low-energy region, while PBEPBE/PBESOL0 performed better for high-energy C–H and O–H stretching modes. The study compared bond lengths and torsion angles between experimental and computational results, revealing variations primarily in the O–H bonds due to the different environments of the molecule in gas-phase and solid-state simulations.

In the Raman spectral simulations, the use of the B3LYP hybrid functional generated marginally better matching spectra than when the PBE-based functionals were used, with the solid-state simulation yielding better results than the single molecule simulation. Discrepancies were observed in the fingerprint region, where the solid-state data matched the experimental data more closely. The modes in the 500–700 cm^–1^ range, attributed to in-plane bending, exhibited good agreement with experimental data in the *CRYSTAL* simulation. The study also highlighted the shifting of peaks in the region of 1450–1550 cm^–1^, corresponding to asymmetric C–H bending, and the role of O–H bending motions in the intensity pattern of this region. *CASTEP*, using plane-wave basis sets, offered comparable agreement with *CRYSTAL* simulations, particularly in the low-energy region.

Infrared spectroscopy focused on high-energy C–H and O–H stretching modes, where PBEPBE/PBESOL0 simulations better aligned with experimental data than B3LYP. Gas-phase simulations in *Gaussian* revealed nine C–H stretching modes, while solid-state simulations in *CRYSTAL* indicated 72 C–H modes due to the presence of eight molecules in the unit cell. A similar pattern was observed for O–H modes, confirming specific vibrations associated with each structural form. Despite significant peak shifting between the simulated and experimental IR spectra, evidence emerged for at least one specific interaction, the O3**(I)**–H–O5**(II)** H-bond, connecting the two distinct molecular structures in the crystal. Additional H-bonding interactions seem possible, but no direct correlation between structure and vibrational data could be established for other such features.

This comprehensive approach underscores the strengths and limitations of both experimental and computational methods in elucidating the vibrational dynamics and intermolecular interactions within the methyl-β-D-ribofuranoside crystal structure. The data sets that were acquired and are reported in this paper are of significant sizes; therefore, only general aspects of what they indicate have been discussed in this work, alongside the more obvious and defining spectral features. Nonetheless, we aim in this study to represent a starting point for further discussions and perspectives on what the various experimental and simulated spectra communicate. Input geometries for the DFT calculations can be improved by using dimer models of the two distinct structures of methyl-β-D-ribofuranoside linked by the H-bonding interaction we have described. The more challenging aspect, ultimately, is to probe the vibrational spectra of this molecule in solution, which is notoriously difficult to achieve at a satisfactory level with computational simulations,^52^ but it is more approachable now that a detailed analysis of the solid-state properties exists. Overall, this analysis contributes valuable insights to the understanding of the structural features and dynamical behavior of methyl-β-D-ribofuranoside.

## Supporting Information Available

A complete description of the experimental custom Raman setup as well as additional computational details, vibrational data, and snapshots of the optimized structures (PDF)
Archive (Optimized_Structure_Files.zip) containing crystallographic information files for the solid-state optimized structures and chemical structure files for the single molecule optimized structures (ZIP)
Archive (DFT_Simulation_Files.zip) containing input and output text files of all the completed calculations, labeled accordingly; for the solid-state simulation in *CASTEP*, only the input file is a text file, the output files being specific. CASTEP files and the Raman data file being a PHONON file; all output files can be opened, and all the associated calculated vibrational modes visualized with the Chemcraft^42^ software, available free of charge online (www.chemcraftprog.com) (ZIP)
