# Supplementary material for: Comprehensive Analysis of Methyl-β-D-ribofuranoside: A Multifaceted Spectroscopic and Theoretical Approach
Source: J Phys Chem A. 2024 Mar 12;128(11):2111–20. doi: 10.1021/acs.jpca.4c00266 (PMC10961842; doi:10.1021/acs.jpca.4c00266)
Supplement: Supplementary file 1 — jp4c00266_si_001.pdf [file jp4c00266_si_001.pdf]

Supplementary Information for:

Comprehensive Analysis of Methyl-β-D-Ribofuranoside: A Multifaceted Spectroscopic and Theoretical Approach

Matei Pascariu[1,2,*], Leonardo Bernasconi[3,*], Matthew Krzystyniak[1], James Taylor[1], and Svemir Rudić[1,*]

1. ISIS Neutron and Muon Source, STFC, Rutherford Appleton Laboratory, Harwell Campus, Oxfordshire, OX11 0QX, UK

2. Department of Chemistry, The University of Manchester, Oxford Road, Manchester, M13 9PL, UK

3. Center for Research Computing & Department of Chemistry, University of Pittsburgh, Pittsburgh, PA 15260, USA

** Authors for correspondence:*

Matei Pascariu – Email: matei.pascariu@stfc.ac.uk

Leonardo Bernasconi – Email: leonardo.bernasconi@pitt.edu

Svemir Rudić – Email: svemir.rudic@stfc.ac.uk

Contents

Custom Renishaw® inVia Raman spectrometer setup S3

Figure S1. The custom Raman centre stick S4

Figure S2. The sample cell S5

Figure S3. Comparison of Raman spectra at different temperatures S6

Additional data for input files (Tables S1 and S2) S7

Optimised structures (Figures S4 to S10) S10

Additional vibrational data (Figures S11 to S13) S13

References S15

Custom Renishaw® inVia Raman spectrometer setup

The Raman spectra were measured with the help of a customised setup which has been previously described [1]. Over the last year, the setup has been improved and additional functionalities (such as temperature control) were added to it; therefore, an up-to-date description is given here. The modified setup consists of a specially designed centre stick (Figure S1) to which a laser probe head fibre-optically coupled to a Renishaw® inVia Raman spectrometer can be attached. This offers a choice of two lasers: a 532 nm, 200 mW, Class 3B, continuous wave, diode-pumped solid-state laser, or a 785 nm, 300 mW, Class 3B, continuous wave, Toptica® diode-pumped solid-state laser.

The Raman centre-stick is comprised of two concentric thin-walled stainless-steel tubes which pass through a vacuum port and a number of thermal radiation/convection shields. With the laser head on top, the stick is approximately 1.65 m in length and is suitable for insertion into a 100 mm-bore cryostat, allowing for measurements at temperatures between 6 K and 350 K. The laser beam has a diameter at the aperture of 700 μm, and it traverses the inside of the inner stainless-steel tube under vacuum. This configuration avoids background scattering from optical fibres, which would have otherwise been in front of the filters in the probe head, and prevents their exposure to cryogenic conditions and large changes in temperature. Using a 20× magnification achromatic lens with a long working distance of 14 mm, the laser beam is focused down to a spot size of approximately 50 μm diameter through a 1 mm thick sapphire window in the lid of the sample cell, which can be attached to the bottom end of the stick, as shown in Figure S1(b). The focusing is adjustable as the lens can be moved vertically from outside of the cryostat through a motorized motion controlled using the Z-drive of an XYZ translation stage (either manually or from within the WiRE™ 4.1 software supplied with the spectrometer). After the light scatters in the sample, the Raman signal returns along the same path.

The laser power is adjustable either in steps using a motorized neutral density filter wheel down to 1% of the maximum power available (~ 3 mW at the sample position) for the 785 nm laser, or using the CoboltMonitor™ 6.0 software designed for the 532 nm laser allowing for fine adjustments to the power in 1 mW increments. Due to low specific heats and poor thermal conductivities, beam-heating effects can be significant at cryogenic temperatures; thus, being able to adjust the power level at the sample position and read precise temperatures is important. For the latter aspect, temperature is recorded both inside the cryostat and on the sample can itself. A platinum resistance temperature detector (RTD) sensor and two wire heaters are attached to the sample as shown in Figure S2(c). The temperature is monitored and controlled through a Lake Shore Model 224 temperature monitor which provides accurate measurements from 1.4 K to 350 K. The time taken to cool from room temperature to the base temperature of the cryostat (3 K for the liquid helium cryostat) is approximately 2.5 hours, although the sample cell never reaches this temperature, settling around 4.5 K, or 6.5 K, with the laser beam on. Overall, the setup allows for Raman scattering measurements at 532 nm and 785 nm with a resolution

of 1 to 4 cm^{-1} over a wide range (0–4000 cm^{-1}) and at cryogenic temperatures matching the INS conditions on TOSCA.

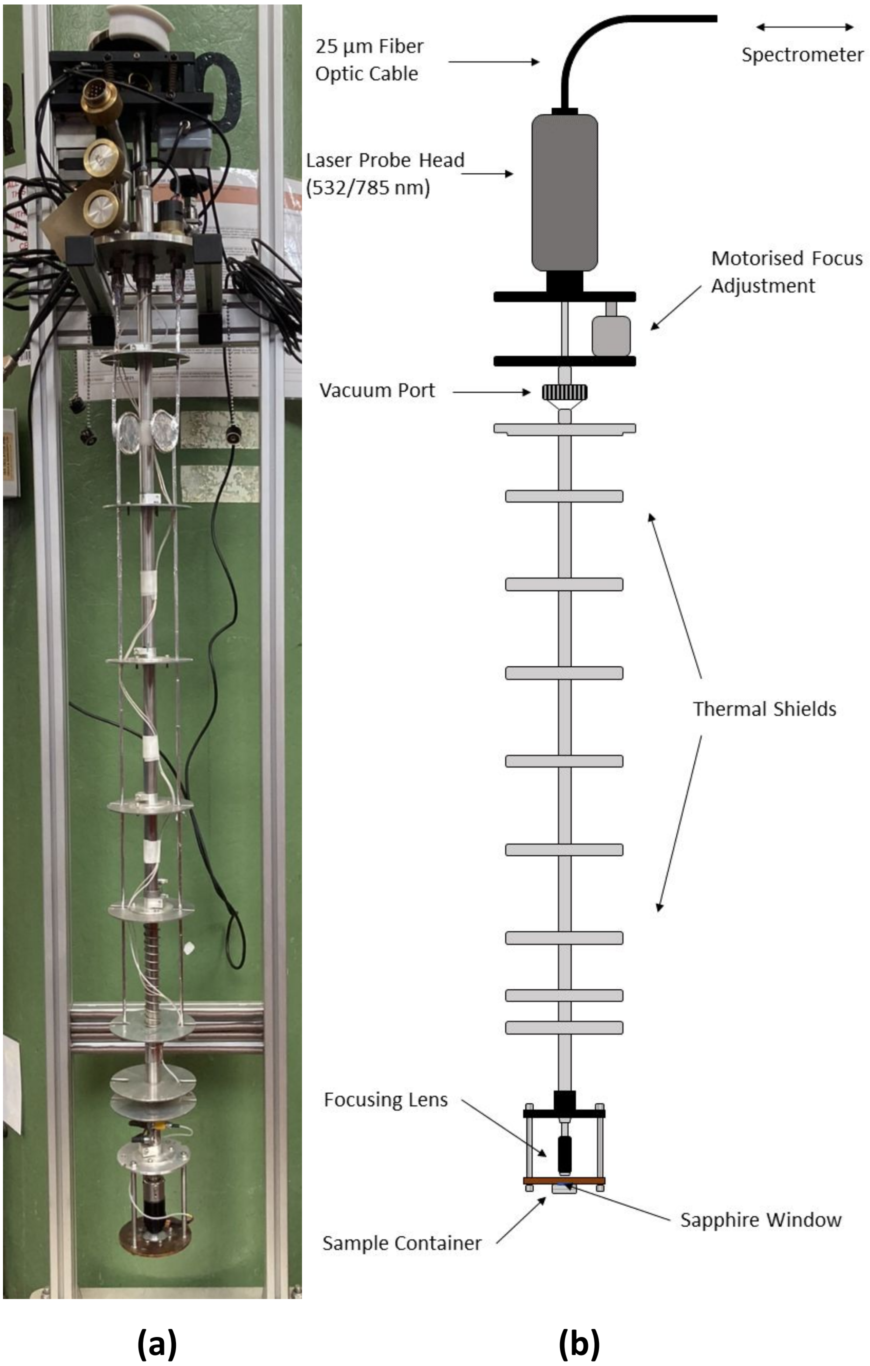

Figure S1. Picture **(a)** and schematic representation **(b)** of the custom Raman centre stick used for the cold Raman measurements.

Raman spectroscopy, in general, does not require large quantities of sample to be used for successful measurements, and this is true for the custom setup presented. The cell can be padded with aluminium foil to allow for as little sample as possible to be loaded (Figure S2(a)). The crucial aspect is for the sample to cover the entirety of the sapphire window (Figure S2(b)) and be in contact with it

to avoid any interference from air molecules (although the vacuum is kept in the sample area throughout the measurement for the same reason) or from the cell material. Indium wire is fitted between the two parts of the cell for better isolation, as the measurement is conducted under a vacuum once the centre stick with the sample is inserted into the cryostat.

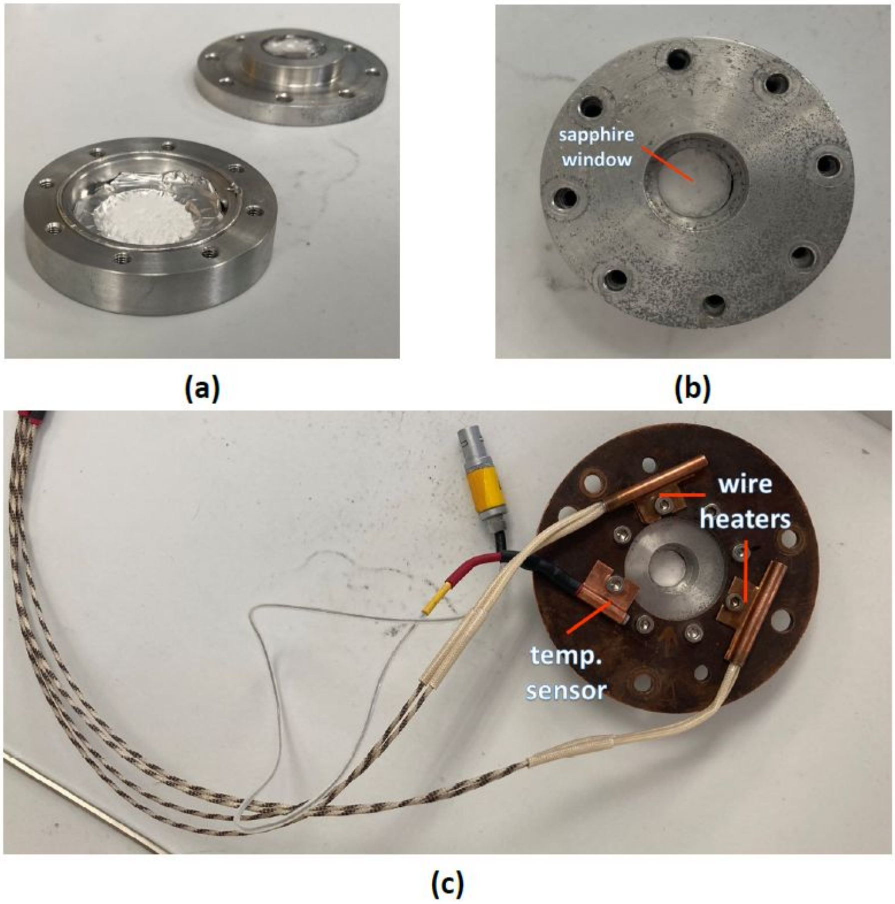

Figure S2. Pictures showing the open cell loaded with sample **(a)**, the closed cell with the sapphire window highlighted **(b)** and the wired (heaters and temperature sensor) copper (Cu) plate secured to the sample cell, ready to be attached to the centre-stick **(c)**.

Raman spectra were also recorded using a commercial benchtop Bruker Senterra confocal Raman microscope setup, equipped with lasers of three different wavelengths: 532 nm, 633 nm, and 785 nm; and capable of measurements of similar resolution (3 to 5 cm^{-1}) and range (40–4000 cm^{-1}). The Raman spectra of methyl-β-D-ribofuranoside were recorded using the 532 nm and 785 nm wavelength lasers at a power of 5.0 mW. Data using the 532 nm laser wavelength was of lower quality, while the 785 nm laser worked better with the methyl-β-D-ribofuranoside sample under the different settings that were tried. Figure S3 shows the comparison between Raman spectra measured with a laser wavelength of 785 nm on both setups at room temperature and a Raman measurement at 6.5 K using the custom setup. It can be seen that the measurements on the modified Raman setup are of the same quality as the spectra

recorded on the research-grade commercial Bruker Raman spectrometer, with the measurement at 6.5 K even providing a better resolution, with sharper peaks, in the region of interest for this study.

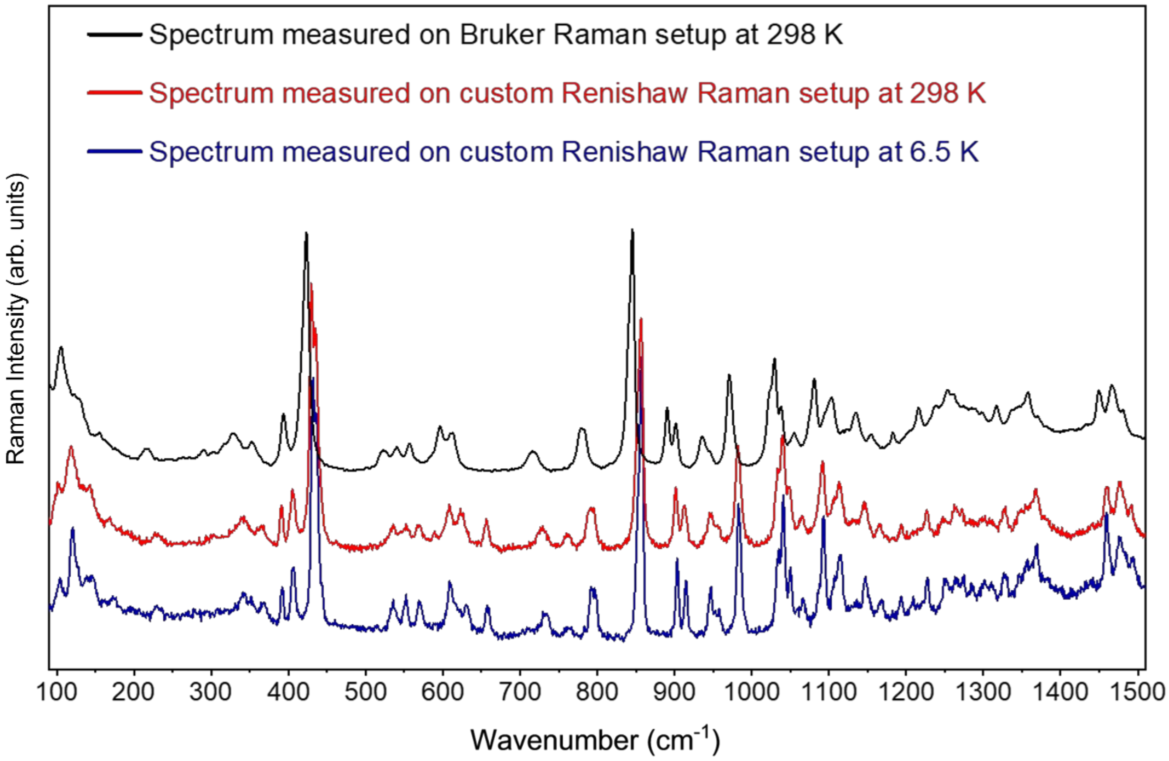

Figure S3. Comparison between Raman spectra of methyl-β-D-ribofuranoside measured at different temperatures and using the two described Raman setups.

Additional data for input files

Table S1. Exponents and contraction coefficients used to define the basis sets for the DFT simulations of methyl-β-D-ribofuranoside with *CRYSTAL 17*.

Atom	Shell	NG[a]	EXP[b]	COE1[c]	COE2[c]
H	S	3	.1873113696D+02	.3349460434D-01	–
			.2825394365D+01	.2347269535D+00	–
			.6401216923D+00	.8137573262D+00	–
	S	1	.1612777588D+00	.1000000000D+01	–
	P	1	.1100000000D+01	.1000000000D+01	–
C	S	6	.3047524880D+04	.1834737130D-02	–
			.4573695180D+03	.1403732280D-01	–
			.1039486850D+03	.6884262220D-01	–
			.2921015530D+02	.2321844430D+00	–
			.9286662960D+01	.4679413480D+00	–
			.3163926960D+01	.3623119850D+00	–
	SP	3	.7868272350D+01	-.1193324200D+00	.6899906660D-01
			.1881288540D+01	-.1608541520D+00	.3164239610D+00
			.5442492580D+00	.1143456440D+01	.7443082910D+00
	SP	1	.1687144782D+00	.1000000000D+01	.1000000000D+01
	D	1	.8000000000D+00	.1000000000D+01	–
O	S	6	.5484671660D+04	.1831074430D-02	–
			.8252349460D+03	.1395017220D-01	–
			.1880469580D+03	.6844507810D-01	–
			.5296450000D+02	.2327143360D+00	–
			.1689757040D+02	.4701928980D+00	–
			.5799635340D+01	.3585208530D+00	–
	SP	3	.1553961625D+02	-.1107775490D+00	.7087426820D-01
			.3599933586D+01	-.1480262620D+00	.3397528390D+00
			.1013761750D+01	.1130767010D+01	.7271585770D+00
	SP	1	.2700058226D+00	.1000000000D+01	.1000000000D+01
	D	1	.8000000000D+00	.1000000000D+01	–

[a] Number of primitive gaussian-type functions (GTF) in the contraction for the atomic orbitals in the shell;
[b] Exponent of the normalised primitive GTF;
[c] Contraction coefficients of the normalised primitive GTF.

Table S2. Cell parameters and fractional atomic coordinates of the asymmetric unit cell of methyl-β-D-ribofuranoside selected from the crystallographic information file (CIF) deposited by C. A. Podlasek *et al.* [2].

**Parameter**	a / Å	b / Å	c / Å	α / °	β / °	γ /°	V / $Å^3$
**Value**	4.8595	24.162	12. 876	90	90	90	1511.84

**Atom**	**Fractional Atomic Coordinates**		
	**x**	**y**	**z**
H	0.408(4)	0.6052(9)	0.160(2)
H	0.160(2)	0.3945(8)	0.312(1)
H	0.842(5)	0.4381(8)	0.523(2)
H	0.196(4)	0.5492(7)	0.252(1)
H	0.304(4)	0.5071(8)	0.172(1)
H	0.701(3)	0.4962(7)	0.266(1)
H	0.202(3)	0.4718(6)	0.367(1)
H	0.426(4)	0.4502(7)	0.521(1)
H	0.707(4)	0.5407(6)	0.520(1)
H	0.458(5)	0.605(1)	0.603(2)
H	0.401(5)	0.6316(9)	0.502(2)
H	0.147(6)	0.616(1)	0.575(2)
H	0.156(5)	0.8765(9)	0.160(2)
H	-0.027(5)	0.7405(8)	0.471(1)
H	-0.275(4)	0.6318(7)	0.362(1)
H	0.341(5)	0.7955(8)	0.194(1)
H	0.270(4)	0.8289(8)	0.304(1)
H	-0.154(4)	0.7855(6)	0.313(1)
H	0.334(3)	0.7243(6)	0.332(1)
H	0.128(4)	0.6390(6)	0.318(1)
H	-0.178(4)	0.6628(7)	0.164(1)
H	0.182(6)	0.7260(9)	0.019(2)
H	0.357(5)	0.674(1)	0.002(2)
H	0.040(5)	0.668(1)	0.006(2)
C	0.3624(3)	0.53481(6)	0.2172(1)
C	0.5438(3)	0.50950(5)	0.2981(1)
C	0.3983(3)	0.46357(5)	0.3580(1)
C	0.5308(3)	0.46724(6)	0.4650(1)
C	0.5625(3)	0.52958(6)	0.4769(1)
C	0.3245(5)	0.60631(7)	0.5489(2)

C	0.1944(3)	0.81350(6)	0.2411(1)
C	-0.0034(3)	0.76973(5)	0.2769(1)
C	0.1384(3)	0.72660(6)	0.3457(1)
C	0.0098(3)	0.67222(5)	0.3100(1)
C	-0.0407(3)	0.68403(5)	0.1960(1)
C	0.1933(6)	0.68544(9)	0.0335(1)
O	0.6237(3)	0.54981(4)	0.37516(7)
O	0.5044(3)	0.57583(4)	0.15866(8)
O	0.4357(2)	0.41226(4)	0.30755(8)
O	0.7895(2)	0.44056(5)	0.46037(8)
O	0.3135(3)	0.55045(5)	0.51296(9)
O	-0.1149(2)	0.74011(4)	0.18960(8)
O	0.0527(3)	0.85481(4)	0.1836(1)
O	0.1176(2)	0.73781(5)	0.45296(8)
O	-0.2416(2)	0.66430(4)	0.36286(9)
O	0.2080(3)	0.67299(5)	0.14201(8)

Optimised structures

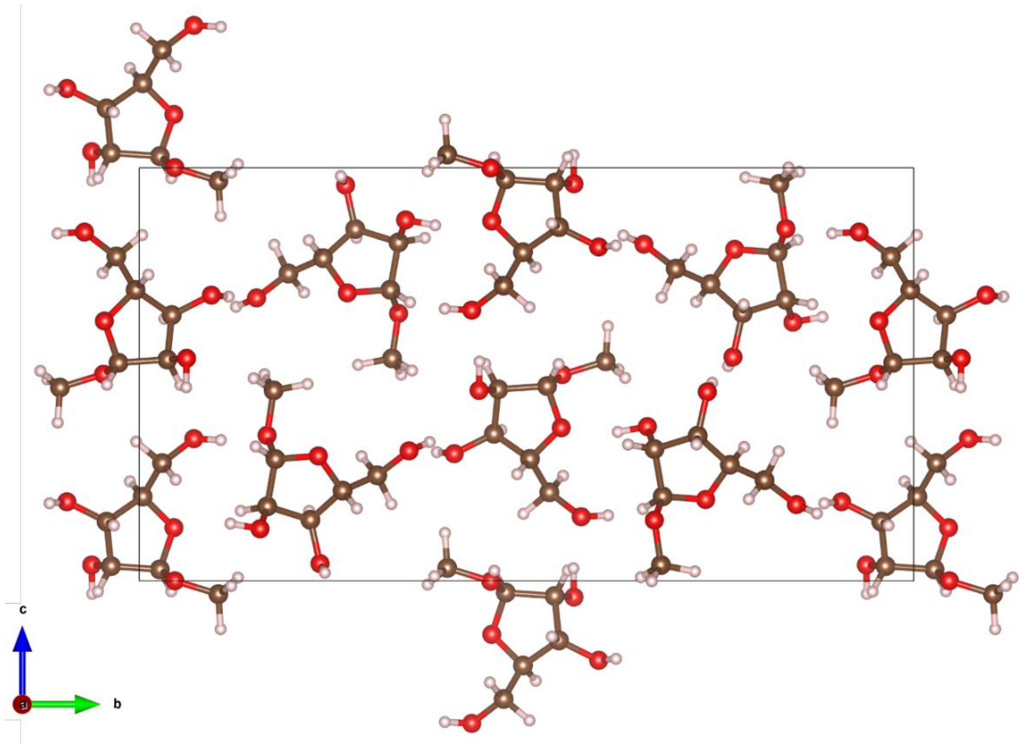

Figure S4. The optimised unit cell of methyl-β-D-ribofuranoside, simulated with *CRYSTAL 17* using the B3LYP hybrid exchange-correlation functional (see CRYSTAL_B3LYP_Optimised_Structure.cif).

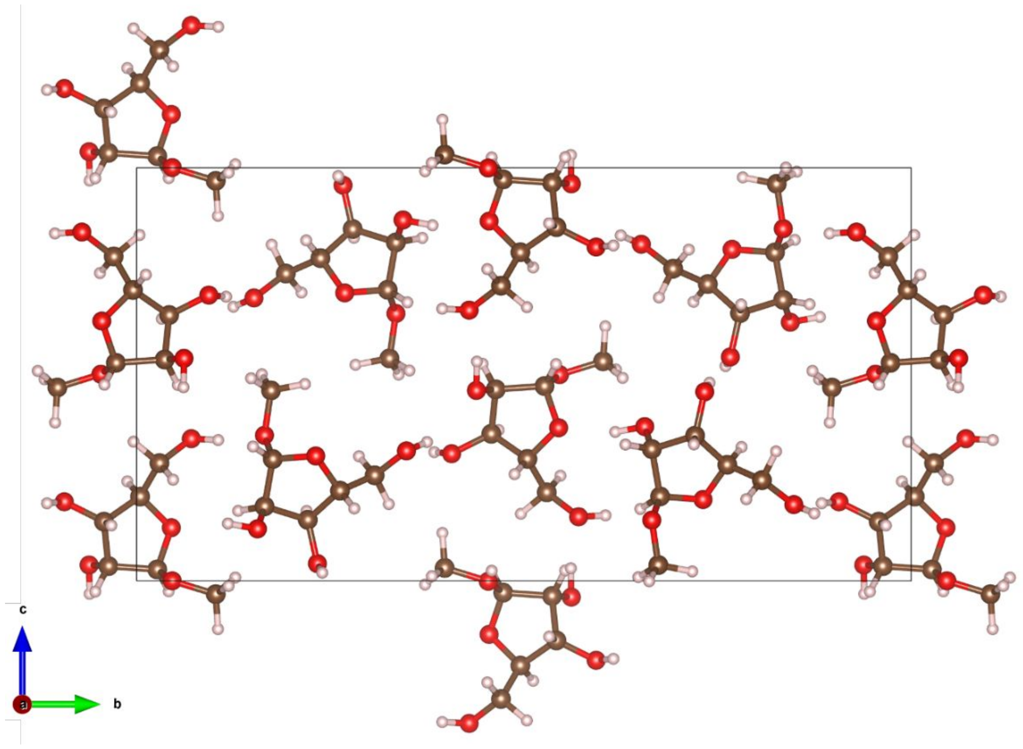

Figure S5. The optimised unit cell of methyl-β-D-ribofuranoside, simulated with *CRYSTAL 17* using the PBESOL0 hybrid exchange-correlation functional (see CRYSTAL_PBESOL0_Optimised_ Structure.cif).

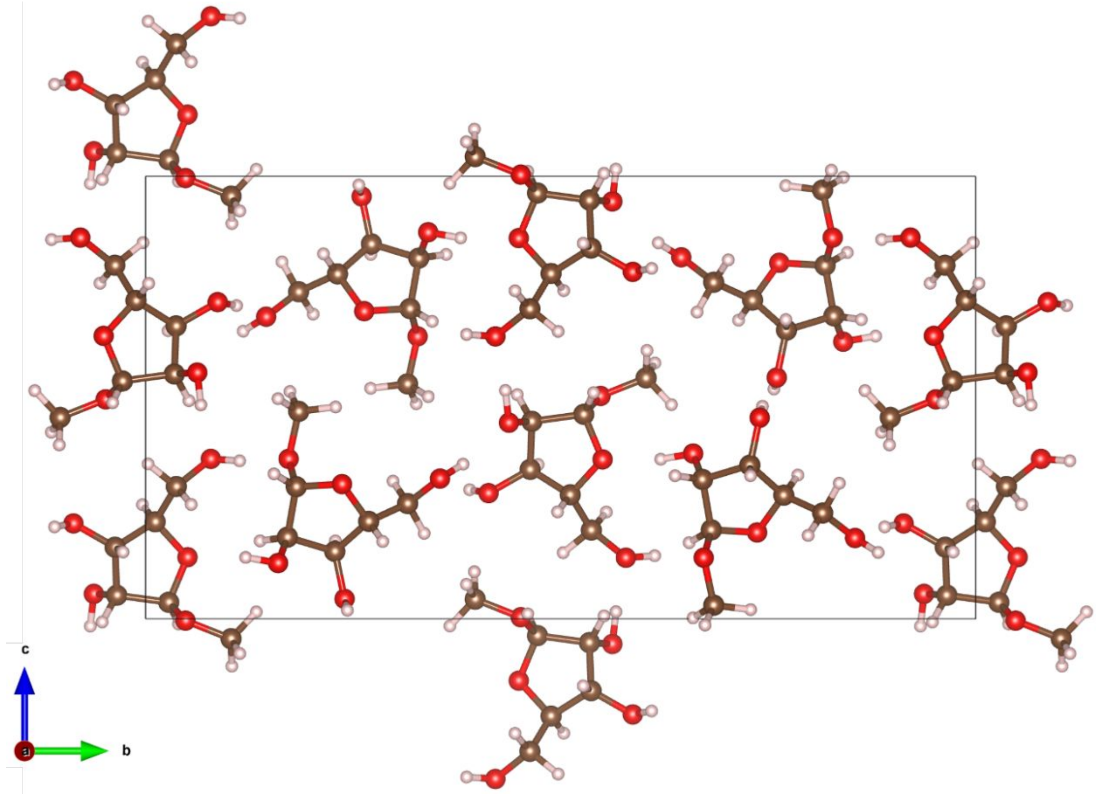

Figure S6. The optimised unit cell of methyl-β-D-ribofuranoside, simulated with *CASTEP 23* using the generalised gradient approximation (GGA) functional PBESOL (see CASTEP_Optimised_Structure.cif).

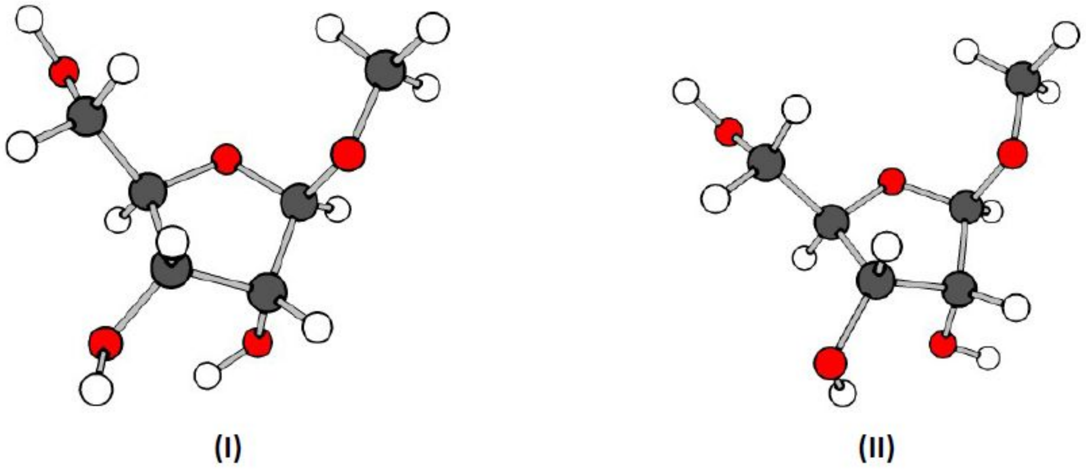

Figure S7. The two structures of methyl-β-D-ribofuranoside optimised in *Gaussian 16* using the B3LYP exchange-correlation functional (see Gaussian_B3LYP_S1_Optimised_Structure.xyz and Gaussian_B3LYP_S2_Optimised_Structure.xyz).

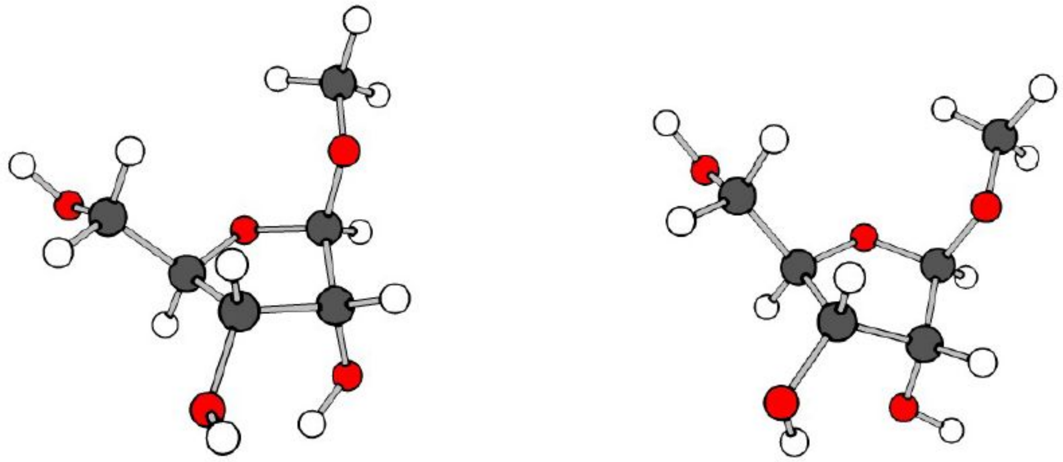

(I) (II)

Figure S8. The two structures of methyl-β-D-ribofuranoside optimised in *Gaussian 16* using the GGA functional PBEPBE (see Gaussian_PBEPBE_S1_Optimised_Structure.xyz and Gaussian_PBEPBE_S2 _Optimised_Structure.xyz).

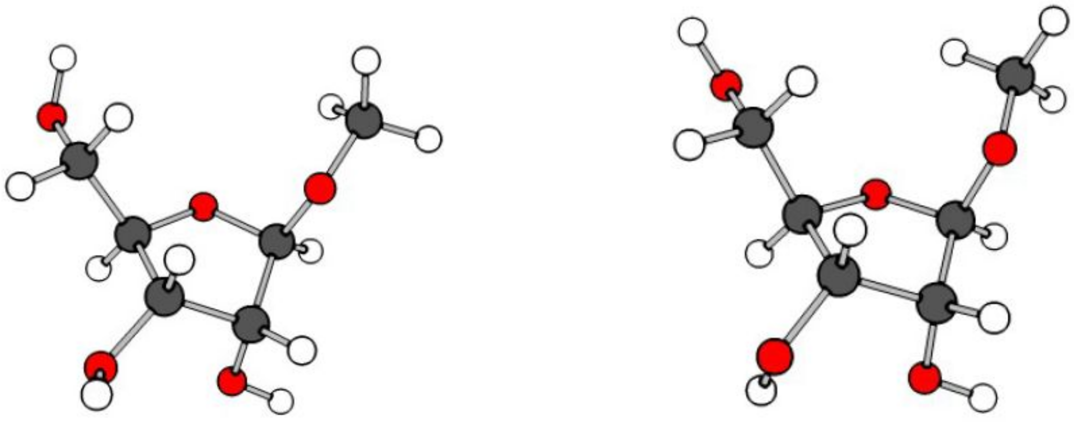

(I) (II)

Figure S9. The two structures of methyl-β-D-ribofuranoside optimised in *CRYSTAL 17* using the B3LYP hybrid exchange-correlation functional (molecules have been manually selected from the optimised unit cell using the same density functional).

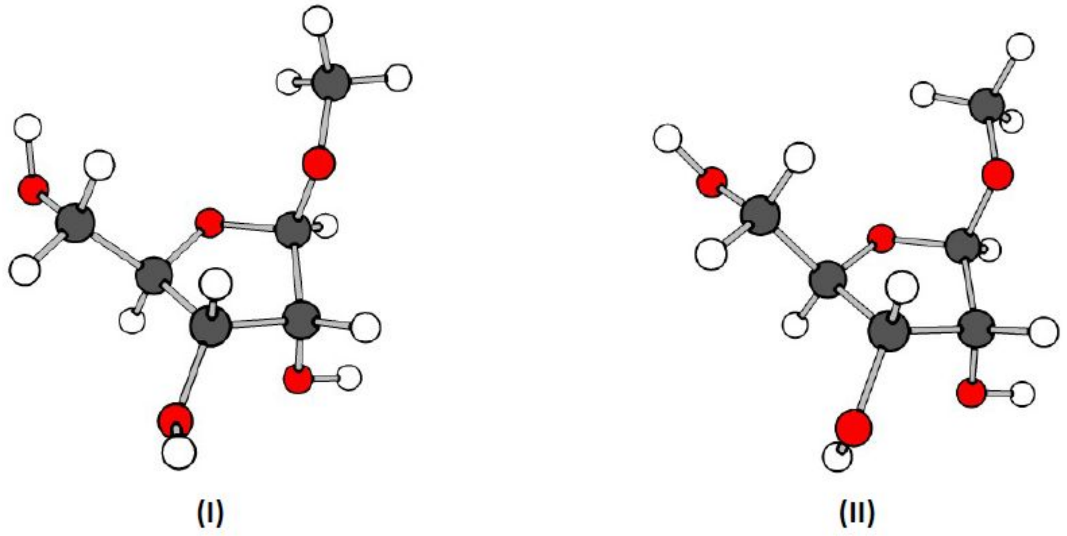

Figure S10. The two structures of methyl-β-D-ribofuranoside optimised in *CRYSTAL 17* using the PBESOL0 hybrid exchange-correlation functional (molecules have been manually selected from the optimised unit cell using the same density functional).

Additional vibrational data

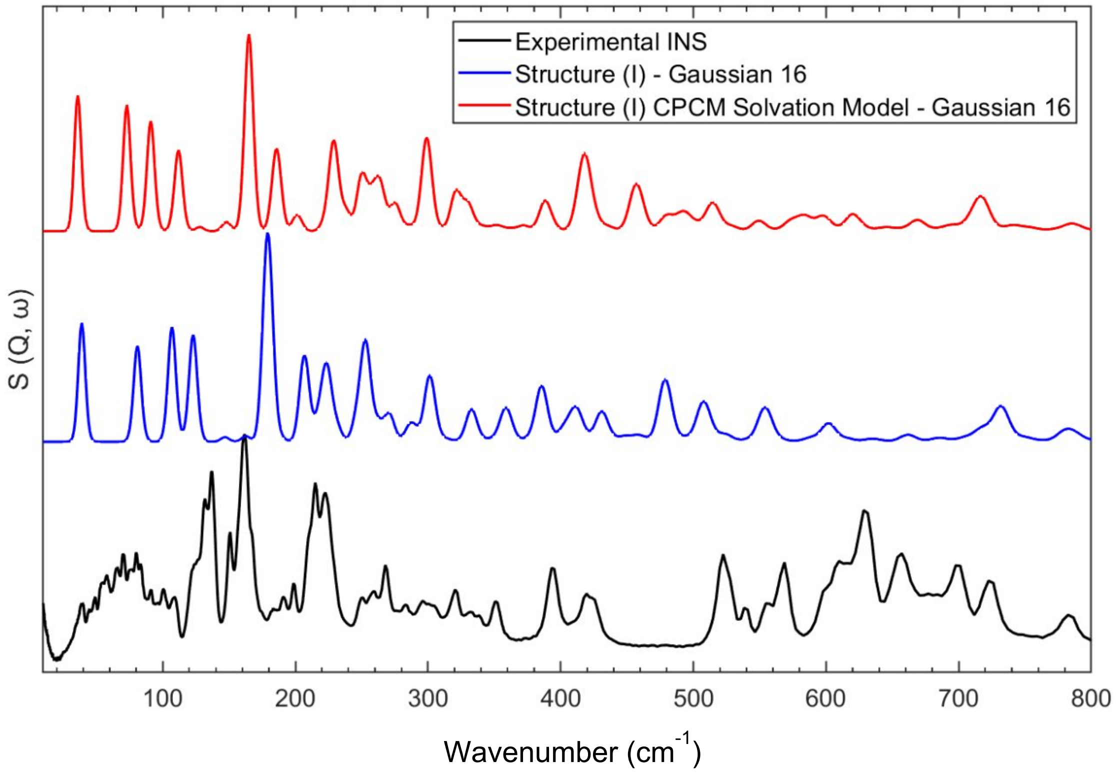

Figure S11. Comparison in the low-energy region of experimental INS spectrum of methyl-β-D-ribofuranoside with theoretical spectra of structure **(I)** of the molecule simulated with *Gaussian* (using the hybrid B3LYP functionals) both with no solvent and with water as a solvent using the CPCM variant of the COSMO solvation model.

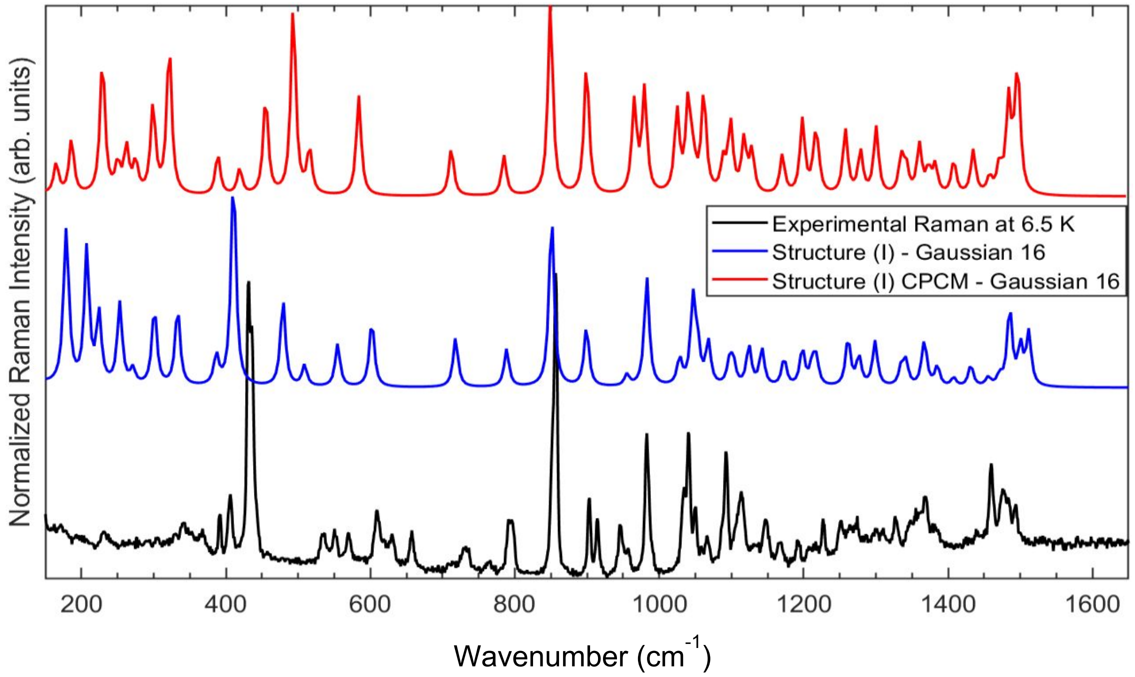

Figure S12. Comparison in the intermediate-energy region of experimental Raman spectrum of methyl-β-D-ribofuranoside with theoretical spectra of structure **(I)** of the molecule simulated with *Gaussian* (using the hybrid B3LYP functionals) both with no solvent and with water as a solvent using the CPCM variant of the COSMO solvation model.

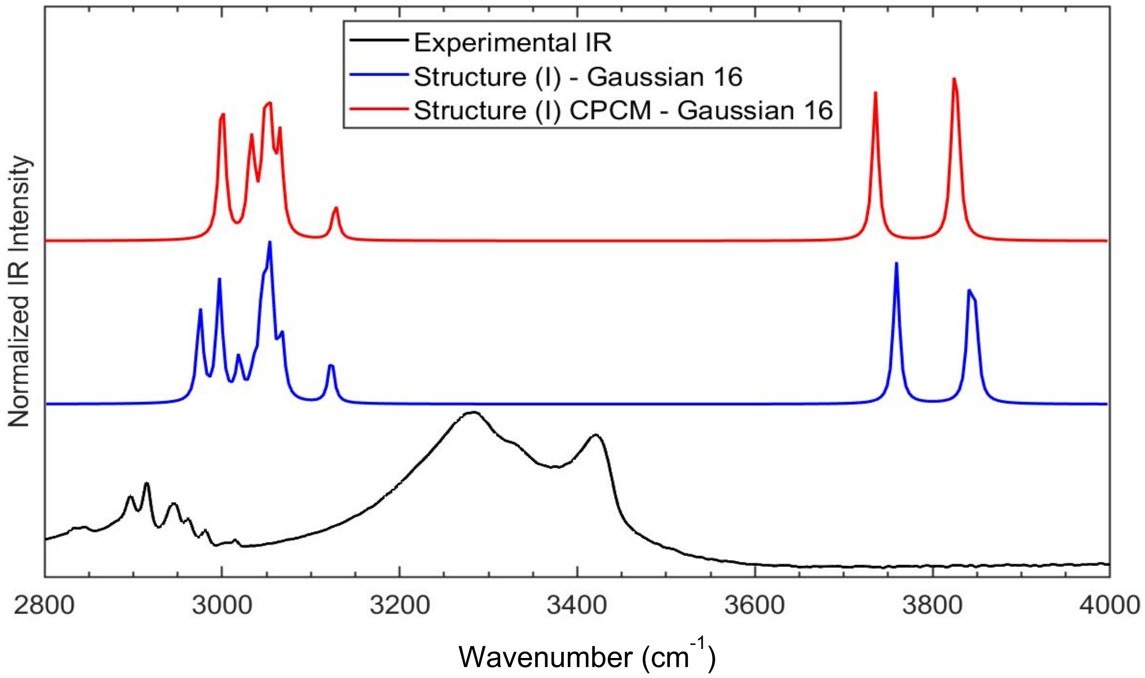

Figure S13. Comparison in the high-energy region of experimental IR spectrum of methyl-β-D-ribofuranoside with theoretical spectra of structure **(I)** of the molecule simulated with *Gaussian* (using the hybrid B3LYP functionals) both with no solvent and with water as a solvent using the CPCM variant of the COSMO solvation model.

References

1. Adams, M. A.; Parker, S. F.; Fernandez-Alonso, F.; Cutler, D. J.; Hodges, C.; King, A. Simultaneous Neutron Scattering and Raman Scattering. *Appl. Spectrosc.* **2009**, *63*, 727–732.
2. Podlasek, C. A.; Stripe, W. A.; Carmichael, I.; Shang, M.; Basu, B.; Serianni, A. S. ^{13}C-^{1}H Spin-Coupling Constants in the β-D-Ribofuranosyl Ring: Effect of Ring Conformation on Coupling Magnitudes. *J. Am. Chem. Soc.* **1996**, *118*, 1413–1425.
